# Dissecting cause and effect in host-microbiome interactions using the combined worm-bug model system

**DOI:** 10.1007/s10522-018-9752-x

**Published:** 2018-03-19

**Authors:** Marina Ezcurra

**Affiliations:** 1 Department of Genetics, Evolution and Environment and Institute of Healthy Ageing, University College London, London, WC1E 6BT UK; 2 School of Biological and Chemical Sciences, Queen Mary University of London, London, E1 4NS UK

**Keywords:** Ageing, Microbiome, Microbiota, *C. elegans*, *E. coli*, *B. subtilis*, Folate, NO, Biofilm, Dysbiosis

## Abstract

High-throughput molecular studies are greatly advancing our knowledge of the human microbiome and its specific role in governing health and disease states. A myriad of ongoing studies aim at identifying links between microbial community disequilibria (dysbiosis) and human diseases. However, due to the inherent complexity and heterogeneity of the human microbiome we need robust experimental models that allow the systematic manipulation of variables to test the multitude of hypotheses arisen from large-scale ‘meta-omic’ projects. The nematode *C. elegans* combined with bacterial models offers an avenue to dissect cause and effect in host-microbiome interactions. This combined model allows the genetic manipulation of both host and microbial genetics and the use of a variety of tools, to identify pathways affecting host health. A number of recent high impact studies have used *C. elegans* to identify microbial pathways affecting ageing and longevity, demonstrating the power of the combined *C. elegans*-bacterial model. Here I will review the current state of the field, what we have learned from using *C. elegans* to study gut microbiome and host interactions, and the potential of using this model system in the future.

## Introduction

Humans coexist in a mutualistic relationship with a diverse gut microbial ecosystem consisting of mainly bacteria, but also viruses, fungi and protozoa. The gut microbiome consists of more than 5 million genes, a genome that is two orders of magnitude than the human genome (Sommer and Bäckhed 2013). The interactions between the host and the microbiota is highly interdependent and it has been suggested that humans should be considered as superorganisms in which the microbial symbionts play essential physiological functions (Fritz et al. 2013). The microbiome can directly affect host physiology by providing food and essential metabolic compounds and inhibiting pathogen colonization by limiting resources available to the invading microbes or competing for attachment sites on the surface of intestinal epithelial cells, or by directly modulating immune system maturation. Conversely, the microbiome has been found to be associated with chronic inflammation (Fritz et al. 2013) and dysbiosis, and imbalance in the microbiome has been linked to diseases such as inflammatory bowel disease, diabetes mellitus, obesity, cardiovascular disease and cancer (Sommer and Bäckhed 2013).

Our current understanding of the human gut microbiota mainly relies on metagenomics profiling. Over the last decade, large-scale metagenomics sequencing studies of human faecal samples have identified many hundreds of species, common sets of microbial species, as well as inter-individual variation. Combined data from the MetaHit and the Human Microbiome Project have provided the most comprehensive view of the composition of the human gut microbiota, identifying a total of 2172 species isolated from human beings, classified into 12 different phyla, of which the large majority belong to the phyla *Proteobacteria*, *Firmicutes*, *Actinobacteria* and *Bacteroidetes* (Schloissnig et al. 2012; Arumugam et al. 2011; Gill et al. 2006; Methé et al. 2012). Further studies have used functional *omic* approaches to attempt to determine which microbial species that are active by identifying microbial proteins and metabolites in faecal samples (Verberkmoes et al. 2009; Gosalbes et al. 2011; Roume et al. 2013; Maurice et al. 2014), and how microbial metabolites are affected in disease states (Jansson et al. 2009). Together these approaches are generating many hypotheses regarding the association between the composition of the gut microbiota and human health.

### The gut microbiota is linked to the ageing process

Considering the many physiological effects of the gut microbiota, it is not surprising that the gut microbiota is also linked to the ageing process of the host. The composition of the gut microbiota changes dramatically during ageing (O’Toole and Jeffery 2015). Healthy gut microbiota is characterised by bacterial taxonomic diversity, while age and frailty is associated with *Bacteroidetes* dominated populations, expansion of more pathogenic bacterial species and a much larger variation between individuals (Claesson et al. 2011; Claesson et al. 2016). Studies of centenarians have shown that their microbiota differs from those of older adults (Biagi et al. 2010; Jeffery et al. 2016), consistent with general age-related microbiota trends.

The numerous metagenomic studies have enabled the definition of a healthy baseline microbiota and generated many hypotheses regarding the association between the composition of the gut microbiota, diseases and the ageing process. These hypotheses include effects on frailty (Claesson et al. 2016), gut health (Biagi et al. 2010), brain plasticity and cognitive function (Leung and Thuret 2015), inflammation (Biagi et al. 2010), metabolic syndrome (Sepp et al. 2014), immunity (Zwielehner et al. 2009) and longevity (Biagi et al. 2010). We now need robust experimental models that allow the systematic manipulation of variables to test the hypotheses arisen from large-scale metagenomic projects and identify the molecular mechanisms underlying the effects of microbial genes on general health and ageing (Fritz et al. 2013).

### Available models to study the effect of the gut microbiota on ageing

Germ-free (GF) mice are the most commonly used animal models to study host-microbe interactions. GF mice are raised in sterile conditions, have no microorganisms living in them and can be made gnotobiotic by colonization with single microbial species, allowing the impact of specific microorganisms to be studied. These kind of studies have shown that the gut microbiota is important for a number of physiological processes in the host including homeostasis, immune system function, development and metabolism (Sommer and Bäckhed 2013). The main challenges with using this model is that maintaining the mice sterile is an expensive and labour intensive challenge, making it a slow, tedious and low-throughput approach. Laboratory mice have a lifespan of approximately 3 years, making the use of gnotobiotic mice in ageing studies even more cost some and challenging (Cho and Blaser 2012).

In addition to mice, zebra fish is being established as a model organism to study ageing and the microbiota, but with a lifespan similar to the mouse lifespan, and tank-based husbandry, it also offers a slow low-throughput approach. Therefore, complementary approaches allowing unbiased high-throughput methods are needed, which can elucidate the mechanisms underlying host-microbiota interactions in vivo (Pham et al. 2008).

The nematode *C. elegans* offers an avenue to dissect the cause and effect by allowing the genetic manipulation of both host and microbial genetics to identify pathways affecting host health. A number of recent studies have identified microbial pathways affecting ageing and longevity in *C. elegans* (Table 1), opening for the possibility to further develop this model to understand the role of the gut microbiota in disease states and ageing. Here I will review the current state of the field, what we have learned so far from using the nematode to study gut microbiome and host interactions, and how *C. elegans* can be further exploited to dissect of complex interactions and mechanisms between the host and its microbiota.

**Table 1:** Publications identifying worm-bug interactions resulting in longevity

Publication	Bacterial species	Bacterial pathway	Host pathway	Physiological effect	Notes
Virk et al. (2012)	*E. coli* OP50, HT115	Folate cycle			
Virk et al. (2016)	*E. coli* BW25113	Folate cycle			
Cabreiro et al. (2013)	*E. coli* OP50	Folate cycle Methionine metabolism (metformin)	AMPK		
Gusarov et al. (2013)	*B. subtilis*		DAF-16, HSF-1	Bacterial gut colonisation, stress resistance?	
Donato et al. (2017)	*B. subtilis* domesticated and undomesticated	Biofilm formation	DAF-16	Bacterial gut colonisation, stress resistance?	Sporulating forms
Smolentseva et al. (2017)	*B. subtilis*	Biofilm formation	MTL-1 (regulated by DAF-16)	Stress resistiance	
Han et al. (2017)	*E. coli* BW25113	Suppression of CA production		Mitochondrial fission, mt UPR, germline tumor suppression, Abeta model suppression	No lifespan effect of CA on OP50
Nakagawa et al. (2016)	*Lactobacillus* LG2055	Unknown	SKN-1 p38 MAPK	Motility, stress resistance, mitochondrial function	
Zhao et al. (2017)	*Bifidobacterium longum* BB60 isolated from cent	Cell wall components	TIR-JNK DAF-16	ND	
Grompone et al. (2012)	*Lactobacillus rhamnosus* CNCM I-3690	ND	DAF-16	Stress resistance Lipid depostion	
	*E. coli* K12	Indole production	AHR-1	No longevity Motility Reproduction	Also in flies
Saiki et al. (2011)	*E. coli* OP50	Respiration		Stress resistance	
Gomez et al. (2012a, b)	*E. coli* OP50	Respiration		Bacterial gut colonisation	
Celhay-Portal (2012)	*E. coli* OP50, Salmonella		DAF-16, DBL-1 (TGF-beta, immunity),	Bacterial gut colonisation	
Podshivalova et al. (2017)	*E. coli* OP50	Unknown		Bacterial gut colonisation	
Youngman et al. (2011)	*E. coli* OP50	Unknown	Innate immunity p38 MAPK	Distension of gut lumen	
Gems and Riddle (2000)	*E. coli* OP50	Unknown			
Garigan et al (2002)	*E. coli* OP50	Unknown			

## The worm-bug: a high throughput model of host-microbiota interactions

The nematode *C. elegans* has many inherent advantages as a model. It has a short life cycle of 3 days, a large brood size and a lifespan of 3 weeks, and is inexpensive and easy to cultivate in the laboratory. *C. elegans* has two sexes: the hermaphrodite, which is self-fertilizing and isogenic, and males, allowing genetic exchange and genetic crosses. *C. elegans* is transparent, allowing imaging of tissues and cells in vivo, without the need of dissection. This model organism also offers many useful tools to dissect complex biological pathways. There are publicly available genetic mutants from the Caenorhabditis Genetics Center, and genome wide RNA interference libraries (Fraser et al. 2000), allowing high-throughput genetic screens. Many additional tools have been developed allowing high-throughput approaches of e.g. survival measurements (Stroustrup et al. 2016; Churgin et al. 2017). The body of *C. elegans* is transparent, allowing the use of fluorescent reporters in vivo. Importantly, *C. elegans* is well-established as an ageing research model and has enabled the identification of pathways influencing ageing, such as the insulin/IGF-1 (IIS) and mTOR pathways, which are evolutionary conserved in mammals (Kenyon 2010). Approximately 50% of the *C. elegans* genes have clear human orthologues, making *C. elegans* ideal to study pathways highly relevant to human biology.

*C. elegans* is a bacterivore, and in the wild *C. elegans* feeds on bacteria that proliferate in decaying organic material, and harbour a diverse bacterial flora in their guts (Schulenburg and Félix 2017). In terms of the viability and evolution of *C. elegans* and its microbes, it is regarded as an indivisible entity, called the “worm-bug” (Cabreiro and Gems 2013). In addition to all the advantages *C. elegans* offers on its own as a laboratory model system, it has been combined with another very successful model, *E. coli*. In the laboratory, *C. elegans* is maintained on a monoxenic culture, typically of the *Escherichia coli* B bacterial strain OP50, using standard microbiology techniques. In addition, other strains such as *Bacillus subtilis* and *E. coli* HT115 are also routinely used (Cabreiro and Gems 2013). The easily obtained control of the gut microbiota in *C. elegans* allows the dissection not only of host pathways, but also of bacterial pathways, and complex interactions between the host and the microbiota. *E. coli* is the most thoroughly studied species on the planet, has well-established genetics and offers many tools such as genome editing, genetic engineering and online databases for analysis of genetic and biochemical functions. Combining the advantages of *E. coli* and *C. elegans* into the worm-bug model system is proving to yield a very powerful combined model system. For example, the *E. coli* Keio deletion collection containing approximately 4000 mutants covering 93% of *E. coli* genes can be used to screen for host phenotypes, and this approach has successfully been used to identify bacterial pathways affecting *C. elegans* ageing (Virk et al. 2016; Han et al. 2017). This approach is not limited *to E. coli*, as there are additional collections in other species (Zhang et al. 2017).

The use of the worm-bug model consisting of a host with a single microbial species as its microbiota offers many advantages in terms of experimental tractability. Furthermore, inexpensive and straight forward cultivation, combined with the short lifespan of *C. elegans*, makes this model system an ideal addition to existing mammalian models to dissect the role of the gut microbiota in human health and ageing. Recent years have seen several high-profile studies using the worm-bug, identifying a number of bacterial and host interactions promoting host longevity.

## Bacterial folate metabolism promotes longevity

One of the most well studied microbial pathway to affect *C. elegans* longevity is folate metabolism. The folate cycle is a series of metabolic steps found in all cells, including both bacteria and animals, required for cell biosynthesis. The products include purines, pyrimidines, glycine, and methionine, and are required to generate the methyl donor molecule S-adenosyl methionine (SAM), which occupies a central role for metabolism of all cells. Folates in their reduced tetrahydrofolate form act as enzymatic cofactors in the folate cycle, and since animals cannot synthesize folates, folates are obtained from their diets and microbiota (Virk et al. 2012).

Disruption of *E.* *coli* folate synthesis promotes longevity in *C. elegans*. Para-aminobenzoic acid (PABA) is a folate precursor. A partial screen of the Keio *E. coli* mutant library identified several mutations in enzymes involved in the synthesis of PABA, *aroD*, *pabaA* and *pabaB*, which increase lifespan in *C. elegans*. Sulfamethoxazole (SMX), a sulfonamide drug that competes with PABA for the active site of the enzyme dihydropteroate synthase, also increases lifespan in *C. elegans*. Reducing folate uptake or metabolism directly in the host does not affect lifespan, showing that lifespan is extended as a result of alterations in bacterial metabolism (Virk et al. 2016; Virk et al. 2012).

Important clues regarding the mechanisms underlying bacterial folate metabolism and host ageing have come from studies using the biguanide metformin, an oral drug widely prescribed to treat type 2 diabetes. The biological activity of metformin goes beyond its antihyperglycemic effects, and has been shown to reduce the risk of cancer (Dowling et al. 2011) and delay ageing in rodents (Anisimov et al. 2011). Metformin also extends lifespan in *C. elegans* (Onken and Driscoll 2010), and this effect is dependent on bacterial activity. In the absence of *E. coli*, or in the presence of dead *E. coli*, metformin is toxic and shortens lifespan. A key player in this bacterial effect is folate metabolism; Metformin does not increase lifespan when mutations in folate cycle enzymes are introduced into *E. coli* and pharmacological inhibition of folate synthesis in *E. coli* mimics metformin treatment. Furthermore, metformin changes the folate composition in *E. coli*, but not in *C. elegans*. Changes in the folate cycle result in perturbations of bacterial methionine metabolism, leading to methionine restriction and activation of the metabolic sensor AMP-activated protein kinase (AMPK) in the host, mimicking molecular features of dietary restriction (Cabreiro et al. 2013).

Collectively these studies show that microbial metabolism can alter ageing in the host, and identify the microbial and host pathways underlying these effects, demonstrating that the worm-bug combined model provides an excellent framework to explore the effects of gut microbiota on ageing. They also raise the question if alterations in the bacterial folate and methionine can mimic dietary restriction and alter ageing in humans as well, and if the gut microbiota can be used for pharmacological interventions.

## Bacterially derived NO enhances host lifespan and stress tolerance

In addition to changes in bacterial metabolism, secreted microbial compounds and signals, such as nitric oxide (NO) have been found to promote longevity in *C. elegans*. NO has many important functions, including affecting neurotransmission, immunity and cardiovascular function by affecting protein function directly and indirectly via post-translational modifications. Although most eukaryotes have endogenous enzymes, NO synthases (NOS), that generate NO, *C. elegans* is unusual in the sense that it has no genes that encode NOS and does not produce NO by NOS-dependent synthesis (Gusarov et al. 2013).

Since NO is a small molecule that diffuses freely across cellular compartments and membranes, NO produced by bacteria can diffuse into worm tissues and affect worm physiology. Rather than using the standard laboratory *E. coli* strain OP50, Gusarov et al. (Gusarov et al. 2013) used *Bacillus subtilis*, a soil bacteria that naturally populates the *C. elegans*’ gut, and produces NO (Gusarov et al. 2013). *B. subtilis* is known to extend lifespan compared to *E. coli* (Garsin 2003), and Gusarov et al. showed that the mechanisms of these longevity effects involve bacterial production of NO in the worm gut, which diffuses to other tissues and initiates a signalling cascade, resulting in a transcriptional response. The transcriptional response involves the longevity genes daf-16, *C. elegans* FOXO which acts downstream of reduced IIS, and *hsf*-*1*, the *C. elegans* heat shock factor 1. DAF-16 is a transcription factor controlling many aspects of physiology and has broad effects on ageing (Kenyon 2010), while HSF-1 mainly acts by regulating the transcription of small heat shock factor proteins. Thus, NO produced by the gut microbiota acts to increase lifespan by activating longevity pathways in the host, providing an example of interspecies signalling by a small molecule.

## Bacterial biofilms promote host health and longevity by reducing IIS

In most laboratory studies, bacteria are grown in shaken-liquid medium, which favours bacteria that grow well in suspension. However, in nature, most bacteria grow in biofilms, that is aggregated to each other and to solid surfaces, and embedded within a slimy extracellular matrix composed of extracellular polymers, including proteins and polysaccharides. Bacteria in biofilms have an unexpectedly high degree of coordinated multi-cellular behaviours that have led to the perception of biofilms as “cities” of microorganisms. In the biofilms bacteria regulate diverse physiological processes and group activities through quorum sensing, a mechanism in which bacterial cells produce, detect and respond to small diffusible signal molecule (Li and Tian 2016).

There is a growing appreciation that, although clearly worthwhile, studies of standard planktonic cultures (single cells floating in liquid) provide us with a biased view of microbial life. The cells in a biofilm are embedded within an extracellular polysaccharide matrix that has been reported to provide the embedded bacteria with protection against a variety of environmental stresses, and interactions among microorganisms in a biofilm enhance nutrient availability and remove toxic metabolites. Thus, bacteria in a biofilm have different characteristics than the same bacteria in isolation (Verhagen et al. 2011).

Two recent studies using *B. subtilis*, a long-serving model for biofilm formation, in combination with *C. elegans*, show that biofilms have beneficial effects on host health and longevity, and shines light on some of the mechanisms involved (Donato et al. 2017; Smolentseva et al. 2017). Donato et al. compared a wild undomesticated strain of *B. subtilis* with the standard domesticated strains of *B. subtilis* and *E. coli*. To promote biofilm formation, they used sporulated forms of *B. subtilis*, which survive the transit through the pharynx and germinate in the worm intestine. The domesticated *B. subtilis* strain increased lifespan and tolerance to different stresses, but all these effects were even more pronounced when fed the undomesticated strain. Why does the undomesticated *B. subtilis* strain extend lifespan compared to the domesticated strain? The authors found that the undomesticated strain colonised the *C. elegans* gut to a larger extent, and that the effect on both gut colonisation and lifespan depended on the ability to form biofilms. Three different strains deficient in the ability to form biofilms due to mutations in *tasA* and *epsG*, which are key components of the extracellular matrix of the biofilm, and in the *bslA* gene which encodes for an essential hydrophobin responsible for the formation of the hydrophobic surface layer that surrounds and protects the biofilm, were tested. The mutations suppressed the longevity effects of the undomesticated strain and reduced gut colonisation compared with the wildtype undomesticated strain, without having sporulation or nutritional effects. Also, the decrease in worm longevity produced by feeding worms each biofilm-defective strain correlated with a decreased bacterial proficiency in gut colonisation (Donato et al. 2017).

Smolentseva et al found similar effects in non-sporulating *B. subtilis*. They first confirmed that non-sporulating *B. subtilis* also form biofilms inside the *C. elegans* gut, and then compared biofilm-producing *B. subtilis* with biofilm-deficient B. subtilis mutants, and showed that production of biofilm results in increased lifespan and resistance to heat, oxidative stress and pathogenic infection, and improved late-life motility. These effects require *mtl*-*1*, a host gene encoding metallothionein, a small cysteine-rich metal-binding protein, which is regulated by DAF-16/FOXO. This study argues that the longevity effects do not require the extracellular matrix itself, but rather that it is the metabolic status of the biofilm producing bacteria that extends lifespan (Smolentseva et al. 2017). Furthermore, the effects of biofilm have been suggested to involve secretion of NO and CSF, a quorum sensing peptide (Donato et al. 2017), but these effects were contradicted by Smolentseva et al (Smolentseva et al. 2017).

Together these studies show that microbial colonisation and biofilms have beneficial effects on host longevity and healthspan, and identify some of the host pathways mediating the responses to the biofilms. The implication of these studies is that bacterial biofilms, and secreted compounds such as NO, act through the classical longevity pathway DAF-16/FOXO and promote longevity by reducing IIS. Further studies will elucidate the details of these interactions and the mechanisms underlying the effect of biofilms on IIS.

## High-throughput approaches uncover bacterial effects on host mitochondria

An important advantage with *C. elegans* is the possibility to use high-throughput approaches to perform large unbiased screens over a range of conditions. Such an approach was used to screen the entire Keio *E. coli* mutant library, covering almost 4000 genes in an *E. coli* K-12 BW25113 background, and resulting in the identification of 29 bacterial genes that increase lifespan (Han et al. 2017). The large majority of these bacterial genes act through well characterised longevity pathways in the host, including the IIS and mTOR signalling pathways, but two of the mutants, ∆*hns* and ∆*lon,* increase lifespan independently of known longevity pathways. *hns* encodes a global DNA-binding transcriptional dual regulator, and *lon* a DNA-binding ATP-dependent protease. Both transcriptional regulators suppress production of colonic acid (CA), a polysaccharide secreted by many enterobacterial species, resulting in high levels of CA secretion in the mutants and longevity in the host. By knocking down a number of *C. elegans* genes and performing imaging and functional analysis, the authors showed that bacterial CA regulates host mitochondrial dynamics. CA is taken up by the host intestine through endocytosis, increases mitochondrial fission in the intestine, acts through mitochondrial respiration and induces transcription of mitochondrial chaperones through mitochondrial UPR (Han et al. 2017).

Supplementation of CA to wildtype bacteria phenocopies the mutant effect not only in *E. coli*, but also in several other bacterial strains. However, CA supplementation does not increase lifespan in the standard laboratory OP50 strain, which is naturally *lon* deficient and already has higher levels of CA, highlighting the importance of the genetic background of bacterial strains on longevity. In contrast to NO, biofilms and folate, the longevity effect of CA does not require live bacteria, meaning that CA alone is enough to exert its beneficial effects and does not act through changes bacterial metabolism (Han et al. 2017).

Why improving intestinal mitochondrial homeostasis increases healthspan and lifespan remains to be explored, but intestinal health has previously been shown to be important for longevity (Ezcurra et al. 2017; Libina et al. 2003), and this study shows how the gut bacteria can act directly to improve intestinal health. The discovery of the influence of CA on host physiology reveals an interesting crosstalk between bacterial signalling and mitochondria in animal hosts. Since CA is produced in response to stress on the part of the bacteria, worms may have evolved response pathways that read bacterial stress in the gut and initiate response pathways as means to offset impending adverse conditions (Gruber and Kennedy 2017).

This study is an excellent example of how the worm-bug offers the possibility of using high-throughput approaches to perform unbiased screens to identify bacterial determinants of late life health, and determine the physiological mechanisms in the host underlying these effects. Importantly, this study goes beyond studying host lifespan, and shows that CA not only increases lifespan but also suppress age-related disease models and delays the onset of muscular mitochondrial fragmentation and locomotor decline. The study also demonstrates how worm-bug can be used as a platform to discover mechanisms conserved in higher organisms, as CA increases lifespan in *Drosophila* and promotes mitochondrial fragmentation in a murine cell line, opening for the possibility that CA could promote healthy ageing in humans.

## Detrimental effects of dysbiosis during ageing

During ageing, changes in the composition of the human gut microbiota take place (Biagi et al. 2012). Dysbiosis has been associated not only with ageing but also with many diseases, including obesity, type 2 diabetes, and arthritis (Sommer and Bäckhed 2013; O’Toole and Jeffery 2015; Cho and Blaser 2012), but the causality between microbial diversity and host health remains unexplored. The mechanisms underlying age-related dysbiosis are likely to be complex and multi-factored, and studies using *C. elegans* offer means to identify microbial and host processes contributing to dysbiosis.

Like humans, *C. elegans* suffers from age-related dysbiosis, and bacterial proliferation of *E. coli* OP50 in the *C. elegans* gut has been implicated as a major contributor of mortality (Gems and Riddle 2000; Garigan et al. 2002; Portal-celhay et al. 2012). During its life course, the relationship between *C. elegans* and *E. coli* changes. In early life, *E. coli* OP50 mainly acts as a food source, and living, metabolically active bacteria are required for normal development and fertility. As the nematodes reach adulthood, *E. coli* OP50 starts proliferating within the gut lumen, and as the animals age, there is a large increase in bacterial load in the gut lumen, accompanied by bacterial packing and constipation (Cabreiro and Gems 2013; Garigan et al. 2002; Portal-celhay et al. 2012; Gomez et al. 2012).

A number of studies and approaches confirm that *E. coli* is pathogenic in late life. Inhibiting bacterial growth by antibiotic treatment increases lifespan (Gems and Riddle 2000; Garigan et al. 2002; Portal-celhay et al. 2012; Podshivalova et al. 2017), and animals with colonised guts have shorter lifespan than animals with non-colonised guts (Podshivalova et al. 2017) suggesting that *E. coli* proliferates and colonises the gut of the host. Furthermore, inhibiting bacterial respiration by introducing mutations in the electron transport chain reduces age-related bacterial proliferation and extends lifespan. This effect is not due to dietary restriction or to compounds released by the bacteria, but rather by lack of bacterial respiration itself, resulting in reduced bacterial growth and suppressed bacterial colonisation of the gut (Saiki et al. 2011; Gomez et al. 2012). Thus, with age, the relationship to *E. coli* changes from being necessary and beneficial to being detrimental and pathogenic, resulting in dysbiosis, colonisation of the gut and mortality. This is not true for all bacterial strains, e.g. the longevity effect of biofilm formation by *B. subtilis* requires bacterial growth, and in this context antibiotics shorten lifespan (Smolentseva et al. 2017). Thus, the effect of bacterial proliferation and dysbiosis is dependent on the bacterial species and its relationship to the host.

## Age-related processes in the host contribute to age-related dysbiosis

The effect of dysbiosis during ageing is also dependent on age-related processes in the host contributing to vulnerability to bacterial proliferation in late life. One such process is immunosenescence, the decline of the immune system that occurs during ageing. *C. elegans* has an innate immune system with evolutionarily conserved signalling; anti-microbial innate immunity is modulated by pathways involving the DAF-16/FOXO transcriptional regulator, the PMK-1 p38 MAP kinase, and DBL-1, homologous to human transforming growth factor b (TGF-b). Knocking down genes in these pathways results in increased bacterial proliferation and decreased host lifespan (Portal-celhay et al. 2012) and there is a strong correlation between immunity decline, increased dysbiosis and mortality. Thus, the ageing process results in decreased efficiency of innate immunity to provide host defence and a loss in the capacity to control bacteria within the gut, and dysbiosis, at least partly regulated by immunosenescence, plays a role in the determination of host lifespan (Portal-celhay et al. 2012).

Other host processes are also involved in determining the effect of bacterial proliferation during ageing. These relate to the decline of tissues that are in direct contract with the gut microbiota, the pharynx and the intestine. The pharynx breaks down bacterial cells during feeding, and age-related decline of the pharynx results in increased bacterial load and bacterial invasion (Garigan et al. 2002; Portal-celhay et al. 2012; Gomez et al. 2012; Zhao et al. 2017). The gut, which digests food and provides a barrier to the external world, also exhibits severe decline during ageing (Herndon et al. 2002), a process that is likely to contribute to increased vulnerability to bacterial pathogens (Youngman et al. 2011). Death is accompanied by an increase of bacterial density in the pharynx and the proximal intestine, but long-lived animals with reduced IIS are protected, and die much later without bacterial colonisation (Podshivalova et al. 2017; Zhao et al. 2017). Collectively, these studies show that ageing and death is accompanied by bacterial proliferation and tissue invasion, showing that dysbiosis plays an important role in *C. elegans* ageing, and that longevity at least in is be due to resistance to bacteria. An interesting twist to these findings is that even though inhibiting bacterial proliferation increases lifespan by 40%, it does not increase healthspan in terms of late-life mobility, and animals treated with antibiotics spend a large section of their life living in decrepitude (Podshivalova et al. 2017). The relationship between between the gut microbiota, host health and ageing is proving to be complex, and there is a need to measure more parameters than just survival when performing ageing studies.

The different host processes contributing to dysbiosis are not necessarily independent. Immunosenescence has been suggested to cause pharyngeal and intestinal decline (Zhao et al. 2017; Youngman et al. 2011), and reduced IIS signalling protects against immunosenescence, pharyngeal and intestinal decline (Garsin 2003; Garigan et al. 2002; Podshivalova et al. 2017; Herndon et al. 2002), supporting the idea that the decline of tissues and immune system are linked. Thus, dysbiosis is likely to be the result of a negative cycle of tissue aging, immunosenescence, and progressive intestinal proliferation of *E. coli* towards the end of life in the host (Youngman et al. 2011).

## Discovering the *C. elegans* native microbiome and its effect on life history

One important question is to what extent studies using laboratory conditions and *E. coli* OP50 reflects the relationship between *C. elegans* and its microbiota in the wild. Decades of studying *C. elegans* in the lab and feeding it with domesticated *E. coli* OP50 has left us with little understanding of how *C. elegans* interacts with its natural microbiota. *E. coli* OP50 was chosen as food source because OP50 forms thinner bacterial lawns, easing visual inspection of *C. elegans*, and due to the availability of OP50 in research laboratories, not because of any resemblance to microbes in the *C. elegans* natural habitats (Samuel et al. 2016). Thus *C. elegans* has been studied in highly artificial laboratory conditions.

Our understanding of *C. elegans* and the relationship to its natural microbiome is now changing as the result of three studies published in 2016. Using partly overlapping and complementary approaches, these studies used next generation sequencing to characterise the native microbiome of *C. elegans*, either in their natural habitats (Samuel et al. 2016; Dirksen et al. 2016), or in microcosms emulating natural habitats (Berg et al. 2016). The identified *C. elegans* microbiome is a rich and diverse microbiome dominated by Proteobacteria such as *Enterobacteriaceae*, Pseudomonas, Stenotrophomans, Ochrobactrum and Sphingomonas, and *Bacteroidetes, Firmicutes*, and differ from the microbial communities in the soil environment, suggesting that host factors influence the microbial composition and that *C. elegans* has a defined, non-random microbiome (Dirksen et al. 2016; Berg et al. 2016). Several additional pieces of evidence support this claim. For example, microbiomes of worms collected from different sampling sites are more similar to each other than to the environmental microbiome (Dirksen et al. 2016; Berg et al. 2016), the *C. elegans* microbiome is distinct compared to the microbiome of closely related nematodes (Berg et al. 2016), and animals collected from the wild maintain their microbiota when cultivated in the lab (Dirksen et al. 2016).

Identification and isolation of the natural microbiome has also allowed the testing of how different species affect host fitness in a laboratory setting. Samuel et al. established culture collections of over 500 wild bacterial strains isolated from the *C. elegans* natural environment, and examined them by growing animals on the individual strains and assaying growth rate and responses to a range of stress and immune reporter genes. 78% of the strains were found to be beneficial, having a higher growth rate and no induction of stress reporters, and 22% to be detrimental, having a slower growth rate and induction of reporters, relative to animals grown on *E. coli* OP50. The effects tended to be genera-specific; several *Proteobacteria*, including *Enterobacteriaceae, Gluconobacter, Enterobacter, Providencia* and most *Lactococcus* strains were beneficial to *C. elegans*. Detrimental genera included Bacteroidetes, such as *Chryseobacterium* and *Sphingobacterium*, and potentially pathogenic *Gammaproteobacteria* (e.g., *Xanthomonas* and *Stenotrophomonas*) (Samuel et al. 2016).

Using a different approach, Dirksen et al. and constructed an experimental microbiome by selecting a subset of 14 isolates representing the most abundant genera of the *C. elegans* microbiome isolated from the wild. Growing animals on the experimental microbiome revealed that bacterial strain frequencies within the gut are distinct from the strain frequencies of the bacterial culture, e.g. *Ochrobactrum* and *Stenootrophomans* isolates was only found as small traces in culture, but formed 20% of the bacterial communities within *C. elegans*, and are likely to be part of the *C. elegans* core microbiome. The experimental microbiome increases population size and enhances worm fitness in comparison to *E. coli* OP50, not only in standard laboratory conditions but also under different stress conditions. Also in this study analysis of individual bacterial isolates identified that the positive effects on population size are likely caused by *Proteobacteria* (Dirksen et al. 2016). Future studies will identify the positive interactions between *Proteobacteria* and *C. elegans.*

The identification of the native gut microbiome opens for many new possibilities using *C. elegans* to understand interactions between the gut microbiome and the host, including aspects shaped by evolution in natural environments, and undetectable in artificial environments using *E. coli* OP50 as the only food source. Considering all the effects on fitness already identified in these three initial studies, it would be surprising if the native gut microbiota also doesn’t also affect healthspan and longevity. Studies of the native gut microbiota are only in its infancy, and coming years will provide us with insight into the role the native the gut microbiota plays in the biology of the host, including ageing.

## Future perspectives

Studies in humans and mice suggest that the gut microbiome plays a role in ageing, but this complex relationship is very difficult to disentangle in higher animals. Recent studies using *C. elegans* combined with bacterial models are starting to provide mechanistic insight into the effects of the gut microbiome on ageing (Table 1). Collectively, they provide with examples of how bacterial metabolism promotes host longevity, including bacterial pathways such as the folate cycle, methionine metabolism, biofilm formation, suppression of CA production and reduction of bacterial respiration. They also strongly suggest that bacterial pathways offer a range of possible interventions to improve late life health in the host.

In addition to microbial pathways, host responses mediating the bacterial effects on longevity have been identified and include conserved longevity pathways, such as DAF-16/FOXO, mTOR and HSF-1 signalling. The activation of longevity pathways by bacteria is not exclusive to the laboratory strains *E. coli* OP50 and *B. subtilis*. Bacterial strains isolated from the human gut, including from human centenarians, have also been shown to activate longevity pathways and promote longevity and health in *C. elegans* (Grompone et al. 2012; Zhao et al. 2017; Nakagawa et al. 2016). The mechanisms by which bacterial signals activate longevity pathways remain to be understood, and will be a considerable challenge for future studies, but it is likely that their activation by microbes could be exploited to improve health and ageing in humans.

One remaining question is if the gut microbiota can promote improved ageing through pathways other than classical longevity pathways. The majority of the recent studies reviewed here have used candidate gene approaches to identify host pathways responding to the microbiome, however *C. elegans* is very well suited for unbiased approaches to identify novel pathways. Replacing candidate gene approaches with screening of mutant and RNAi libraries will allow identification of novel ways by which the host responds to the bacteria. This will be further helped by new automated approaches to measure lifespan and healthspan. Recently several automated systems have been developed, including the Lifespan Machine, where the survival rates of large populations of animals are monitored using flatbed scanners (Stroustrup et al. 2016), and the WormMotel, a microfabricated device for long-term cultivation and automated longitudinal imaging of large numbers of individual animals (Churgin et al. 2017). These and other similar systems have been developed to perform automated lifespan and healtshpan experiments with minimum manual input, and can be combined with existing scalable automation technology such worm sorters, robotic plate handlers, and chemical library screening tools, allowing the study of many bacterial species and pathways and host genotypes. Automated approaches also include behavioural tracking which enable high-throughput measurements of healthspan. For example, The WormMotel allows the measurement not only of lifespan, but also of behavioural decline in individual animals. Another study used the Multi-Worm Tracker computer-vision system (Swierczek et al. 2011) to measure stimulated locomotion and study end-of-life decrepitude (Podshivalova et al. 2017). *C. elegans* ageing studies have traditionally been focused on measuring lifespan, and the addition of healthspan measurements adds an important dimension to our understanding of ageing.

The biggest remaining question is what physiological effects the gut microbiota generates in the host. Most studies have focused on lifespan effects without looking at how tissues and cells are affected. Using a different approach, (Han et al. 2017) identified effects resulting from bacterial CA production involving mitochondrial fragmentation and mtUPR in the intestine, the tissue in direct contact with the microbiota, and beneficial effects in other tissues, including a germline tumor progression model. Also (Zhao et al. 2017; Podshivalova et al. 2017) looked at the effect of bacteria in tissues and showed that bacterial invasion of the pharynx and the intestine occurs during ageing and correlates with death. The continued and expanded use of physiologically relevant approaches will shine light on the mechanisms through which bacterial pathways act to improve late life health.

## Conclusions

It is becoming clear that the microbiome is linked to human health. Current studies show that microbial populations appear in specific disease states and during senescence, and the challenge now is to establish if there is a causal link between microbial variation, disease and ageing. This requires robust experimental models that allow the systematic manipulation of variables to test the multitude of hypotheses arisen during recent years. We are only just starting to identify how bacterial pathways affect host biology and ageing, and the factors shaping the composition of the gut microbiota. Combining the nematode *C. elegans* with bacterial model organisms allows approaches that cannot easily be used in mammalian systems, such as large-scale unbiased screens, automated approaches and in vivo imaging in combination with genomics/genetics and metabolomics, allowing the dissection of complex interactions and mechanisms between the host and its microbiota. The potential for this area of research is immense and during the years to come will see further understanding of complex host-microbiota interactions.
